# Reviewer Acknowledgement 2013

**DOI:** 10.1186/1471-2377-14-28

**Published:** 2014-03-11

**Authors:** Tim Shipley

**Affiliations:** 1 BioMed Central, Floor 6, 236 Gray's Inn Road, London WC1X 8HB, UK

## Abstract

**Contributing reviewers:**

The editors of BMC Neurology would like to thank all our reviewers who have contributed to the journal in Volume 13 (2013).

## 

Jan O Aasly

Norway

Susan Abushakra

United States of America

Charalampos Agakidis

Greece

Vered Aharonson

Israel

Wasim Ahmad

Pakistan

Kiril Aleksov Ahtarovski

Denmark

Vladeta Ajdacic-Gross

Switzerland

Saeed Akhtar

Kuwait

Uazman Alam

United Kingdom

Dimitrios Alexopoulos

Greece

Jens Allendoerfer

Germany

Mohammed Almekhlafi

Canada

Pierre Amarenco

France

Maria Amato

Italy

Nada Andelic

Norway

Corrado Angelini

Italy

Angelo Antonini

Italy

Brian Appleby

United States of America

Adria Arboix

Spain

Larissa Arning

Germany

Willem Arts

Netherlands

J. Wesson Ashford

United States of America

Elsa Azevedo

Portugal

Jean Philippe Azulay

France

Tobias Back

Germany

Jonathan Baker

United States of America

Inês Baldeiras

Portugal

Sevket Balta

Turkey

Yoram Barak

Israel

Iacopo Barbetta

Italy

Stefano Bastianello

Italy

Grant Bateman

Australia

Spyros Batzios

Greece

Peter Bede

Ireland

Annemerle Beerthuizen

Netherlands

Uwe Beffert

United States of America

David Begley

United Kingdom

Christina Bell

United States of America

Ajediran Bello

Ghana

Ralph Benedict

United States of America

Julian Benito-Leon

Spain

Roberto Bergamaschi

Italy

Joseph Berger

United States of America

Robert Bermel

United States of America

Jacqueline Bernard

United States of America

Julie Bernhardt

Australia

Marcelo Berthier

Spain

Antonio Bertolotto

Italy

Peter Bingham

United States of America

Patrizia Bisiacchi

Italy

Frederic Blanc

France

Miguel Blanco

Spain

Angeliki Bogosian

United Kingdom

Samuel Bolitho

Australia

Sevasti Bostantjopoulou

Greece

James Bowen

United States of America

Francesco Brigo

Italy

Fabienne Brilot

Australia

Aldobrando Broccolini

Italy

Jacoline Bromberg

Netherlands

Gila Bronner

Israel

Kolbjørn S Brønnick

Norway

Benjamin Rix Brooks

United States of America

Jared Bruce

United States of America

John CM Brust

United States of America

Brian Buck

Canada

Philip Buck

United States of America

Jean-Marc Bugnicourt

France

Jean-Marc Burgunder

Switzerland

Ted Burns

United States of America

Norberto Cabral

Brazil

Dominique Anne-Michele Cadilhac

Australia

Jianfang Cai

China

Petra Callenbach

Netherlands

Michele Callisaya

Australia

Bruce Campbell

Australia

Patrícia Canhão

Portugal

Fredric Cantor

United States of America

Bingzhen Cao

China

Marco Capobianco

Italy

Roberto Cappellani

United States of America

Rafael Carel

Israel

Ilaria Casetta

Italy

Mirela Cerghet

United States of America

Rute Cerqueira

Portugal

Pavel Chalupa

Czech Republic

Bernard Chan

Singapore

Richard Chan

Canada

Jong Hee Chang

Korea, South

Andreas Charidimou

United Kingdom

Jack Chen

United States of America

Ping-Kun Chen

Taiwan

Harvinder Singh Chhabra

India

Adriano Chio

Italy

Yao-Chung Chuang

Taiwan

Daniel Ciampi de Andrade

Brazil

Claudio Cimminiello

Italy

David Clarke

Canada

Tim Clarner

Germany

Eleonora Cocco

Italy

Angela Colantonio

Canada

Anne Comi

United States of America

Diane Cookfair

United States of America

Gianluca Coppola

Italy

Pedro Cougo-Pinto

Brazil

Athanasios Covanis

Greece

Helen Cross

United Kingdom

Wenguo CUI

China

William J Culpepper

United States of America

Armin Curt

Switzerland

Sami Curtze

Finland

Anna Czlonkowska

Poland

Raffaele D'Amelio

Italy

Stephane Darteyre

France

Mamede de Carvalho

Portugal

Elvira Valeria De Marco

Italy

Jean de Oliveira

Brazil

Boel De Paepe

Belgium

Sandrine de Ribaupierre

Canada

Jerome de Seze

France

Marina de Tommaso

Italy

David Decker

United States of America

Pilar Delgado

Spain

Vida Demarin

United States of America

Athena Demertzi

Belgium

Chantal Depondt

Belgium

Massimiliano Di Filippo

Italy

Alessio Di Fonzo

Italy

Marco DiBonaventura

United States of America

Maria Cristina Digilio

Italy

Mazen Dimachkie

United States of America

Thorsten Doeppner

Germany

Ondrej Dolezal

United Kingdom

Rosario Donato

Italy

Ville Dorothee

France

Alexander Dressel

Germany

Peter Drummond

Australia

Andrew Ducruet

United States of America

Hugues Duffau

France

Tomasz Dziedzic

Poland

Alan Eckeli

Brazil

Juergen Eggers

Germany

Irina Elovaara

Finland

Ann-Kristin Gunnes Elvrum

Norway

Ilka Engelmann

France

Ricardo Erazo

Chile

Nima Etminan

Germany

Ann Faerden

Norway

Cristian Falup-Pecurariu

Romania

Yuhua Fan

China

Ana Faustino

Portugal

Kai Feng

China

Shejun Feng

China

Peter Ferenci

Austria

Oscar Fernández

Spain

Israel Fernandez Cadenas

Spain

José L. Fernández-Torre

Spain

Pasquale Ferrante

Jamaica

Mark Ferro

Canada

Sydney Finegold

United States of America

Clare Fowler

United Kingdom

Diego Franciotta

Italy

Leonora Franke

Germany

Karen Fritchie

United States of America

Jong-Ling Fuh

Taiwan

Tereza Gabelic

United States of America

Aurelia Gaeta

Italy

Alberto Gajofatto

Italy

Elvira Galarraga

Mexico

Salvatore Galati

Switzerland

Shawn Gale

United States of America

Juan Gálvez-Acebal

Spain

James Galvin

United States of America

Xavier Game

France

Ziv Gan-Or

Israel

Chunfang Gao

China

Nicolas Gaspard

United States of America

Charly Gaul

Germany

Thomas Geeraerts

France

Mathias Gelderblom

Germany

Adriana Georgescu

Romania

Angelo Ghezzi

Italy

Daniele Ghezzi

Italy

Emily Gilmore

United States of America

Stefan Gold

Germany

Todd Goldberg

United States of America

Samuel Goldman

United States of America

John Gommans

New Zealand

Francisco Gondim

United States of America

Carlos Gordon

Israel

Paul H Gordon

France

Dido Green

United Kingdom

Joel Greenspan

United States of America

Judith Greer

Australia

Milica Gregoric Kramberger

Slovenia

Wolfgang Grisold

Austria

Caspar Grond-Ginsbach

Germany

Bradley Gross

United States of America

Kirsten Gruis

United States of America

Zezong Gu

United States of America

Eric Guedj

France

Orlando Guntinas-Lichius

Germany

Tanya Gurevich

Israel

Gunter Hoeglinger

Germany

Mario Habek

Croatia

Monika Hackl

Austria

Georgios Hadjigeorgiou

Greece

Shaheryar Hafeez

United States of America

Michal Haran

Israel

Todd Hardy

Australia

James Harrop

United States of America

Rowan Harwood

United Kingdom

Steven Hass

United States of America

Masaharu Hata

Japan

W Allen Hauser

United States of America

Christopher Hawkes

United Kingdom

Daichi Hayashi

United States of America

Kentaro Hayashi

Japan

Yukiko Hayashi

Japan

Jian He

China

Brianna Heggeseth

United States of America

Ingo Helbig

Germany

Andrew Henderson

Australia

Paul Hewson

United Kingdom

Akiyuki Hiraga

Japan

Amber Hogart Begtrup

United States of America

Timothy Hohman

United States of America

Trygve Holmoy

Norway

Dana Horakova

Czech Republic

Patrizia Hrelia

Italy

Ching-Lin Hsieh

Taiwan

Sung-Tsang Hsieh

Taiwan

Chenya Huang

Hong Kong

Hanneke Hulst

Netherlands

Aatif Husain

United States of America

Michael Hutchinson

Ireland

Ing-Shiou Hwang

Taiwan

Philip Iffland

United States of America

Takahiro Iizuka

Japan

Caio Imaizumi

Brazil

Domenico Intiso

Italy

Marco Iosa

Italy

Masaki Ito

Jamaica

Ifeanyi Iwuchukwu

United States of America

Sandra Jacobson

United States of America

Andreas Jager

Germany

Amteshwar jaggi

India

Roland Dominic Jamora

Philippines

Douglas Jeffery

United States of America

Jiann-Shing Jeng

Taiwan

Jesper Jensen

Denmark

Beom S. Jeon

Korea, South

Seul-Ki Jeong

Korea, South

Djordje Jevtovic

Serbia

Qian Jia

China

Hao Jiang

China

Wei-Jian Jiang

China

Glen Jickling

United States of America

Sheng Chih Jin

United States of America

Yan Jin

United States of America

Mikko Joensuu

Finland

Gareth John

United States of America

Katarina Jood

Sweden

Wolfgang Jost

Germany

Csaba Juhasz

United States of America

Simon Jung

Switzerland

Carme Junque

Spain

Santoshi Kamei

Japan

Masahiro Kamouchi

Japan

Kyusik Kang

Korea, South

Osamu Kano

Japan

Kai Kappert

Germany

Andreas Karabinis

Greece

Ioannis Karakis

United States of America

Symon Kariuki

Kenya

Jan Kassubek

Germany

Naoki Kawaguchi

Japan

Paula Kersten

United Kingdom

Paula Kersten

New Zealand

Gokhan Keser

Turkey

Dheeraj Khurana

India

Alice Kiger

United Kingdom

Hee Tae Kim

Korea, South

Jong Kim

Korea, South

Kazumi Kimura

Japan

Markus Kipp

Germany

Jun-ichi Kira

Japan

Adam Kirto

Canada

Zelma Kiss

Canada

Ilya Kister

United States of America

Pim Klarenbeek

Netherlands

Timothy Kleinig

Australia

Jan-Helge Klingler

Germany

Thomas Klockgether

Germany

Giacomo Koch

Italy

Sebastian Koch

United States of America

Bobby Koeleman

Netherlands

Boyd Koffman

United States of America

Channa Kolb

United States of America

Genevieve Konopka

United States of America

Marianne Eline Kooi

Netherlands

Milind Kothari

United States of America

Fernando Kowacs

Brazil

Izumi Koyanagi

Japan

Michal Král

Czech Republic

Jörg Kraus

Austria

Christine Kremer

Sweden

John Kriesel

United States of America

Venkatesh Krishnamurthy

Australia

Jerzy Krupinski

Spain

Wojciech Kulak

Poland

Andrea Kübler

Germany

Sudhir Kumar

India

Akira Kunimatsu

Japan

Kalle Kurppa

Finland

Hyung-Min Kwon

Korea, South

Peter Langhorne

United Kingdom

Paul Langley

United States of America

Jeanne Latourelle

United States of America

Kate Laver

Australia

Seung-Jae Lee

Korea, South

Peter Leigh

United Kingdom

Jon Levine

United States of America

Michael Levy

United States of America

Jan Lewerenz

Germany

Simon Lewis

Australia

James Lewsey

United Kingdom

Ming Lim

United Kingdom

Shen-Yang Lim

Malaysia

Richard Lindley

Australia

Jiing-Feng Lirng

Taiwan

Giancarlo Logroscino

Italy

Francesco Lolli

Italy

Armando López-Guillermo

Spain

Iraj Lotfinia

Iran

Martin Lotze

Germany

Linda Lowes

United States of America

Cheng-Hsien Lu

Taiwan

Po-Haong Lu

United States of America

Pei Lin Lua

Malaysia

Lih-Fen Lue

United States of America

Codrin Lungu

United States of America

Sheng Luo

United States of America

Mauricio Machado

Brazil

Amir Hadi Maghzi

Iran

Parker Magin

Australia

Romy Mahrer Imhof

Switzerland

Christoph Maier

Germany

Johannes Mair

Austria

Bik Ling Man

Hong Kong

Marcello Mancini

Italy

Aikaterini Markopoulou

United States of America

Ruth Ann Marrie

Canada

Elisabeth Marsh

United States of America

Paula Martin

United States of America

Vittorio Martinelli

Italy

Kenia Martínez

Spain

Sergi Martinez-Ramirez

United States of America

Sheryl Martin-Schild

United States of America

Ayrton Massaro

Brazil

Flavia Mattioli

Italy

Johannes Mayr

Austria

Patrick McDonald

Canada

Ian McKeith

United Kingdom

Pierre Mégevand

United States of America

Bijal Mehta

United States of America

Vincent Meininger

France

Omer Mekki

Kenya

Mario O. Melcon

Argentina

George Mellick

Australia

Monika Merkes

Australia

Sarlota Mesaros

Serbia

Patrick Meybohm

Germany

Slawomir Michalak

Poland

Haralampos Milionis

Greece

Styliani Milioti

Greece

Alireza Minagar

United States of America

Pablo Mir

Spain

Tristan Mirault

France

Masahiro Mishina

Japan

UshaKant Misra

India

Paige Moorhouse

Canada

Neil Morgan

United Kingdom

Dominik Morhard

Germany

Reg Morris

United Kingdom

Keith Muir

United Kingdom

Anjana Munshi

India

Hiroyuki Murai

Japan

Lorina Naci

Canada

Kazuma Nakagawa

United States of America

Makoto Nakajima

Japan

Taizen Nakase

Japan

Osvaldo J M Nascimento

Brazil

Wassim Nasreddine

Lebanon

Kiumarss Nasseri

United States of America

Manuela Neuman

Canada

Huu Phuc Nguyen

Germany

Danielle Ni Chroinin

Ireland

Shekoufeh Nikfar

Iran

Ichizo Nishino

Japan

Eduardo Nobile-Orazio

Italy

Satoru Noguchi

Japan

Masahiro Nomoto

Japan

Kathleen Norman

Canada

Anne Louise Oaklander

Afghanistan

Mark Obermann

Germany

Laszlo Olah

Hungary

Javier Olazarán

Spain

Robert Olson

Canada

Hiroshi Ooba

Japan

Francesco Orzi

Italy

Damjan Osredkar

Norway

Mehdi Oualha

France

Linda Oude Griep

United Kingdom

Betul Ozdilek

Turkey

Natalia Palacios

United States of America

Prakash Paliwal

Singapore

Pier Paolo Panciani

Italy

Shree Pandya

United States of America

Peter Panegyres

Australia

George Papadimas

Greece

Kwang Yeol Park

Korea, South

Jonathon Parker

United States of America

Antonio Pascotto

Italy

Francesco Patti

Italy

Friedemann Paul

Germany

Elena Pegoraro

Italy

Francesco Pelliccia

Italy

Chava Peretz

Israel

Santiago Perez-Lloret

France

Joseph Pergolizzi

United States of America

Amy Perrin Ross

United States of America

alessandro pezzini

Italy

Elmar Hans Pinkhardt

Germany

Erik Pioro

United States of America

David Pitt

United States of America

Mara Popovic

Slovenia

Emilio Portaccio

Italy

Ron Postuma

Canada

William Potter

United States of America

Luca Prosperini

Italy

Harald Prüss

Germany

Josep Puig

Spain

Vinod Puri

India

Francisco Purroy

Spain

Jukka Putaala

Finland

Bin Qin

China

Michael Rafii

United States of America

Sreeram Ramagopalan

United Kingdom

Pedro Ramos-Cabrer

Spain

Wendy Raskind

United States of America

Chaturbhuj Rathore

India

Miriam F Reelick

Netherlands

Mathew Reeves

United States of America

Irena Rektorova

Czech Republic

Norman Relkin

United States of America

Jason Richardson

United States of America

Fred Rincon

United States of America

John Ringman

United States of America

Pablo Ripollés Vidal

Spain

James Riviello

United States of America

Reuben Robbins

United States of America

Annie Rochette

Canada

Andrea Romigi

Italy

Tom Roques

United Kingdom

Steffen Rosahl

Germany

Eric Rosenthal

United States of America

Lewis P. Rowland

United States of America

Elisa Rubino

Italy

Clodagh Ryan

Canada

Katharina S Sunnerhagen

Sweden

Lidia Sabater

Spain

Simona Sacco

Italy

Carsten Saft

Germany

Mohammad Ali Sahraian

Iran

Arnaud Saj

Switzerland

Stephen Salloway

United States of America

Jacinda Sampson

United States of America

Alvaro Sanabria

Colombia

Friedhelm Sandbrink

United States of America

Filippo M. Santorelli

Italy

Ferdinando Sartucci

Italy

Jaume Sastre-Garriga

Spain

Giovanni Savettieri

Italy

Antonio Scalfari

United Kingdom

Fabien Scalzo

United States of America

Jan Friedrich Scheitz

Germany

Franz Schelling

Austria

Yitzhak Schiller

Israel

Ilana Schlesinger

Israel

Arlene Schmid

United States of America

Ludger Schöls

Germany

Menno Schoonheim

Netherlands

Christoph Schrader

Germany

Monika Schulte-Altedorneburg

Germany

Corina Schuster

Switzerland

Carolyn Schwartz

United States of America

Thomas Scott

United States of America

John Seibyl

United States of America

Kathleen Shannon

United States of America

Eilon Shany

Israel

Arvind Sharma

India

Manish Sharma

United States of America

Geoffrey Sheean

United States of America

Dean Sherzai

United States of America

Jun Shimizu

Japan

Won-Seob Shin

Korea, South

Ishwar Bahadur shrestha

Nepal

Shanop Shuangshoti

Thailand

Michael Shy

United States of America

Gabriele Siciliano

Italy

Alcino Silva

United States of America

Nicholas Silvestri

United States of America

Mauro Silvestrini

Italy

Balwinder Singh

United States of America

Marc Sitbon

France

Mark Slevin

United Kingdom

Ale Smidts

Netherlands

Lauri Soinne

Finland

Dimitrios Sokolis

Greece

Jose Solis

Spain

Sarah Song

United States of America

Heather Spader

United States of America

Gabriela Spulber

Sweden

Stella Stabouli

Greece

Sonja Ständer

Germany

Joel Stein

United States of America

Israel Steiner

Israel

Fabrizio Stocchi

Italy

Jon Stone

United Kingdom

Lars Jacob Stovner

Norway

Pasquale Striano

Italy

Yasuo Sugita

Japan

Arias Susana

Spain

Sigurd Süssmuth

Germany

Shigeaki Suzuki

Japan

Yasushi Takagi

Japan

Kiyohisa Takahashi

Japan

Federica Tamburella

Italy

Lan Tan

China

Norio Tanahashi

Japan

Lucia Tancredi

Italy

Sung-Chun Tang

Taiwan

Christian Tanislav

Germany

Marc Tardieu

France

Wichittra Tassaneeyakul

Thailand

Jeanne Teitelbaum

Canada

Devin Terhune

United Kingdom

Charlotte Teunissen

Netherlands

Serge Thal

Germany

Vincent Thijs

Belgium

Steven Thijsen

Netherlands

Gretchen Tietjen

United States of America

Eric Ting

Singapore

Mathias Toft

Norway

Hiroyuki Tomiyama

Japan

Mehmet Topcuoglu

United States of America

L Gerard Toussaint III

United States of America

Vladimir Trajkovic

Serbia

Daniel Truong

United States of America

Victor Tse

United States of America

Lyn Turkstra

United States of America

R. Scott Turner

United States of America

Antonino Tuttolomondo

Italy

Kayihan Uluc

Turkey

Roberto Vagnozzi

Italy

Joseph Valls-Sole

Spain

Peter Van den Bergh

Belgium

Aad van der Lugt

Netherlands

Erik van Duijn

Netherlands

Helene Van Ettinger-Veenstra

Sweden

Neeltje van Haren

Netherlands

Erwin van Wegen

Netherlands

David Vance

United States of America

Euthymia Vargiami

Greece

Ildikó Vastagh

Hungary

Carmine Vecchione

Italy

László Vécsei

Hungary

Fausto Viader

France

Leo Visser

Netherlands

Anand Viswanathan

United States of America

Bettina von Sarnowski

Germany

Rolf Wachter

Germany

Amanda Waddell

United States of America

Mark Wagshul

United States of America

Hiroki Wakabayashi

Japan

Douglas Walker

United States of America

Harrison Walker

United States of America

Allan Walkey

United States of America

Lei Wan

Taiwan

Jin-Hui Wang

China

Michel Weijerman

Netherlands

Bianca Weinstock-Guttman

United States of America

David Werring

United Kingdom

J Bradley White

United States of America

Roland Wiest

Switzerland

Manfred Windisch

Austria

John Winer

United Kingdom

Gavin Winston

United Kingdom

Karin Wirdefeldt

Sweden

Bryan Woodruff

United States of America

Tzu-Ching Wu

United States of America

Zhi-Ying Wu

China

Yunyun Xiong

China

Zeyu Xiong

United States of America

Ichiro Yabe

Japan

Vijayshree Yadav

United States of America

Yasumasa Yamamoto

Japan

Satoshi Yamashita

Japan

GUo-Yuan Yang

China

Songran Yang

China

E. Ann Yeh

United States of America

Leonard Yeo

Singapore

Bryan Young

Canada

Shengyuan Yu

China

Hon Yuen

United States of America

Burak Yulug

Turkey

Dimitrios Zafeiriou

Greece

Paolo Zamboni

Italy

Scott Zamvil

United States of America

Dong Zhao

China

Jay-Jiguang Zhu

United States of America

Robert Zivadinov

United States of America

Maja Zivkovic

Serbia

## Pre-publication history

The pre-publication history for this paper can be accessed here:

http://www.biomedcentral.com/1471-2377/14/28/prepub

